# Surgical Options for the Refractive Correction of Keratoconus: Myth or Reality

**DOI:** 10.1155/2017/7589816

**Published:** 2017-12-18

**Authors:** L. Fernández-Vega-Cueto, V. Romano, R. Zaldivar, C. H. Gordillo, F. Aiello, D. Madrid-Costa, J. F. Alfonso

**Affiliations:** ^1^Fernández-Vega Ophthalmological Institute, Oviedo, Spain; ^2^Department of Corneal and External Eye Diseases, The Royal Liverpool University Hospital, Liverpool, UK; ^3^Instituto Zaldivar, Mendoza, Argentina; ^4^Department of Corneal Disease, Instituto Zaldivar, Mendoza, Argentina; ^5^Moorfields Eye Hospital, London, UK; ^6^Optics II Department, Optics and Optometry Faculty, Complutense University of Madrid, Madrid, Spain

## Abstract

Keratoconus provides a decrease of quality of life to the patients who suffer from it. The treatment used as well as the method to correct the refractive error of these patients may influence on the impact of the disease on their quality of life. The purpose of this review is to describe the evidence about the conservative surgical treatment for keratoconus aiming to therapeutic and refractive effect. The visual rehabilitation for keratoconic corneas requires addressing three concerns: halting the ectatic process, improving corneal shape, and minimizing the residual refractive error. Cross-linking can halt the disease progression, intrastromal corneal ring segments can improve the corneal shape and hence the visual quality and reduce the refractive error, PRK can correct mild-moderate refractive error, and intraocular lenses can correct from low to high refractive error associated with keratoconus. Any of these surgical options can be performed alone or combined with the other techniques depending on what the case requires. Although it could be considered that the surgical option for the refracto-therapeutic treatment of the keratoconus is a reality, controlled, randomized studies with larger cohorts and longer follow-up periods are needed to determine which refractive procedure and/or sequence are most suitable for each case.

## 1. Introduction

Keratoconus is a bilateral chronic, asymmetric, progressive ectatic condition, in which the cornea assumes a conical shape that induces abnormalities such as high regular and irregular astigmatism and increased higher order aberrations (HOAs), with adverse effects on the visual quality of the patients concerned [1, 2]. It is well established that keratoconus appears in the adolescence and its progression is more acute up to the third decade of life [2]. A recent study shows that the estimated prevalence of keratoconus was 265 cases per 100,000 [3]. This study also showed that the prevalence was higher in men than in women [3]. Keratoconus provides a decrease of quality of life to the patients who suffer from it [4]. Moreover, it has been reported that despite its low prevalence and that it unusually leads to blindness, its impact on the public health is much higher than expected [5].

The method used to correct the refractive error of these patients may also influence on the impact of the disease on their quality of life. It has been reported that rigid gas permeable contact lenses (RPGCLs) minimize the impact of the keratoconus on the quality of life of the patients compared with spectacles [6]. Perhaps, the higher corrected distance visual acuity (CDVA) with RPGCL and the spectacle independency could explain these findings. Although keratoconus was historically considered as a noninflammatory condition, recent researchers revealed increased levels of inflammatory mediators in tears of keratoconus patients without clinical signs of inflammation [7]. Hence, it has been suggested that keratoconus should not be classified as a noninflammatory disorder and even that the pathogenesis of keratoconus progression could include chronic inflammatory events [8, 9]. It should be noted that contact lens wear is intrinsically inflammatory [10]. Lema et al. [11] reported that wearing RPGCL provides an increased level of proinflammatory mediators in the tears of patients with keratoconus. So, the question here is whether it is possible to improve the quality of life of keratoconus patients without using CLs or spectacles.

de Freitas Santos Paranhos et al. [12] reported that intrastromal corneal ring segment (ICRS) implantation not only improved the visual quality of the keratoconus patients, but also had a positive impact on their quality of life. However, in this study, it was not reported whether after ICRS implantation the patients were fitted with contact lenses or spectacle correction.

Generally, the visual rehabilitation for keratoconic corneas requires addressing three concerns: halting the ectatic process, improving corneal shape, and minimizing the residual refractive error. The treatment would depend, among other factors, on each of these concerns and its influence on the disease and quality of vision of the patient. That is, keratoconus is progressing or not, the cornea shape is very irregular with a high level of higher order aberrations (HOAs) and poor CDVA or not, and finally the degree of associated ametropia. The purpose of this review is to describe the evidence about the conservative surgical treatment for keratoconus aiming to therapeutic and refractive effect. We have not included the keratoplasty in this review, which is the surgical procedure for advanced keratoconus. The preoperative clinical features, the complexity of the procedure, and the potential long-term complications make the objectives of this procedure go beyond refractive ones.

## 2. No Combined Procedure

### 2.1. Excimer Laser Surgery

Most of the studies of corneal refractive surgery in keratoconus are in combination with other treatments, which aim to stabilize the disease (these studies will be discussed below). In order to carry out the literature search, we used the following databases: PubMed (United States National Library of Medicine), Web of Science (Thomson Reuters), Embase (Reed Elsevier Properties SA), and Scopus (Elsevier). The search used a combination of the following keywords: (keratoconus OR keratoconic) AND (PRK OR LASIK OR excimer laser surgery). The search was limited to English language publications and peer-reviewed scientific reports. We found 13 articles, which met with our inclusion criteria, that is, corneal refractive surgery for the treatment of the refractive correction in keratoconus. We excluded the studies in which the corneal refractive surgery was combined with other techniques. In most of these publications, the level of scientific evidence of the studies was IV (according to the National Health and Medical Research Council guidelines for interventional studies [13]). This level represents the case series studies with preoutcomes and postoutcomes, and it provides the lowest level of scientific evidence.

There was only one report, which studied the treatment keratoconus with laser in situ keratomileusis (LASIK) [14]. Patients with moderate or mild keratoconus, stable refraction for at least 2 years, and age ranged from 31 to 74 years were analysed. Only 16 eyes were studied, and 3 eyes required keratoplasty after LASIK. In addition, there were eyes that lost visual acuity over the follow-up period, suggesting that the ectasia was progressing.

Another important question about this article is whether all cases included were really keratoconus. This paper was published in 1999, and it is likely that some cases diagnosed as mild keratoconus, with the current advanced technology, would not have been diagnosed as keratoconus, a false positive. In summary, it seems that LASIK should not be considered for patients with keratoconus. Several studies [15–24] have reported the outcomes of photorefractive keratectomy (PRK) in keratoconus suspects, fruste form or mild-moderate keratoconus. In all studies, the disease was stable at the moment of the surgery. Mortensen and Ohrstrom in 1994 [15] treated with PRK five keratoconus that were going to undergo PKP. In four eyes, there was a reduction of the astigmatism and an increase of the visual acuity, avoiding the need for PKP. The authors confirmed these results in a study published 4 years later, with a larger sample and longer follow-up in primary keratoconus [16]. In this study, none of the eyes showed a disease progression after PRK. Sun et al. [17], in a retrospective study, compared the refractive outcomes after PRK in 5 keratoconus-suspected eyes with those in healthy eyes. The refractive outcomes were comparable in both groups. However, it is important to note that only 5 keratoconus-suspected eyes were analysed. Two more recent papers studied the long-term results of PRK in keratoconus suspects [18, 19]. In the first one [18], 12 keratoconus-suspected eyes of 6 patients were included and the follow-up was 4 years. The second one [19] analysed 62 eyes of 42 patients; the mean of follow-up was 4.8 ± 1.4 years. Both studies concluded that PRK in eyes with suspected keratoconus, carefully selected, might be a safe and effective procedure for reducing or eliminating myopia and/or astigmatism. Two studies evaluated the PRK in patients with mild to moderate stable keratoconus [20, 21]. Chelala et al. [20] in a five-year follow-up study analysed the visual outcomes of PRK in 119 eyes with grade 1-2 keratoconus according to Amsler-Krumeich classification. 79 eyes (66.3%) had an uncorrected distance visual acuity (UDVA) of 20/20 at 5 years of follow-up. Only two eyes (1.7%) showed progression of the disease at 5 years of follow-up. In the Khakshoor et al. [21] study, 38 stable keratoconus (grades I-II, Amsler-Krumeich) of 21 patients over 40 years old were recruited. At the last follow-up visit, in 22 eyes, the UDVA was 20/20 and in 8 eyes 20/25. These authors suggested that a residual central corneal thickness higher than 450 *μ*m seems to be sufficient to prevent the disease progression. Kasparova and Kasparov [22] combined PRK with phototherapeutic keratotectomy (PTK) to treat primary keratoconus. They reported good visual and refractive outcomes, although progression of the disease was reported in six eyes (8.6%). Finally, topography-guided [25] and wavefront-guided PRK [26] appears to be safe and effective to reduce the corneal aberrations and improve the visual quality in keratoconus suspects and mild to moderate keratoconus.

The thresholds of the ablation amount and residual corneal thickness are a key point for a safe ablation. In Bilgihan et al.'s study [18], the residual stroma was thicker than 400 *μ*m in all eyes at the completion of stromal ablation. Kasparova and Kasparov [22] recommended a minimal corneal thickness > 500 *μ*m and a residual corneal thickness ≥ 450 *μ*m. Koller et al. [23], Chelala et al. [20], and Guedj et al. [19], in their respective studies, also kept a minimum residual corneal thickness of ≥450 *μ*m. Khakshoor et al. [21] in a group of keratoconic patients with a mean age of 44 years old reported safe outcomes maintaining a residual corneal thickness ≥ 400 *μ*m. The authors suggested that, as the cornea is much more stiffness in older patients due to natural collagen cross-linking, the residual corneal thickness can be thinner.

From these studies, it seems that PRK in mild to moderate stable keratoconus might be an effective procedure for improving UDVA in patients with mild refractive errors. Despite these encouraging results of PRK, it should be taken with caution. Firstly, the evidence level of the studies was low; most of the studies were conducted in the 1990s or early 2000s. Some of these studies included keratoconus suspects or early keratoconus. Maybe, with the current technological advances, some of these cases would have been classified as healthy eyes (false positives).

### 2.2. Phakic Intraocular Lenses (pIOLs)

Phakic intraocular lens can be implanted either in the anterior chamber (AC pIOL) or in the posterior chamber (PC pIOL). Both offer correction of high spherical and cylinder errors. The literature search was using the terms “(keratoconus OR keratoconic) AND (ICL OR implantable Collamer OR phakic intraocular lens)” in the following databases: PubMed, Web of Science, Embase, and Scopus. We limited the search to English language and peer-reviewed publications. We found 18 articles, which met with our inclusion criteria, that is, phakic intraocular lens implantation in keratoconus. We excluded the studies in which this procedure was combined with other techniques. Six articles studied AC pIOLs [27–32] and 9 PC pIOLs [33–41], and 1 was a comparative study between both [42].  Table 1 summarizes the main visual and refractive outcomes reported after AC pIOL. The reduction in refractive error is accompanied by a great improvement in UDVA, where data are available, and in CDVA (range 0.08 to 0.2 decimal scale). Overall, the studies suggest good visual and refractive outcomes. However, it should be noted that the number of cases analysed in these studies was low and the largest series included 36 eyes. Furthermore, it is well known that one of the main complications with AC pIOL is the endothelial loss [43]; hence, further studies with more cases and longer term follow-up should be carried out to assess the safety of this procedure in keratoconus patients. In a retrospective study comparing the visual and refractive outcomes of AC and PC pIOLs, Alió et al. [42] reported that both modalities are a suitable refractive surgical option for stable keratoconus.  Table 2 summarizes the visual and refractive outcomes after PC pIOLs. According to these studies, more than 70% of the cases, the spherical equivalent is within ±1.00 D of the emmetropia after PC pIOL. The reduction in a refractive error is accompanied by a great improvement in UDVA.

The majority of reported complications after ICL implantation are cataract formation [44]. Guber et al. [45] reported the rates of cataract formation 10 years after PC pIOL implantation. The study included 133 eyes of 78 patients. Cataract surgery was performed in 18 eyes at 10 years after PC pIOL implantation. Alfonso et al. [46] retrospectively analysed the prevalence of cataract after PC pIOL implantation. The study included 3240 eyes. The authors reported that the incidence of cataract was low after PC pIOL implantation at the 6-year follow-up. They found that the rate of cataract was higher in patients with a high refractive error.

A suitably sized PC pIOL can prevent the alteration of the anterior segment structures. An oversized PC pIOL can provide pupillary block, while an undersized lens increases the risk for cataract development. Boxer Wachler and Vicente [40] conducted a comparative study in which they used two methods to select the length of PC pIOL in keratoconus patients. One based on white-to-white distance and the other on sulcus-to-sulcus distance. The authors found that both methods provide adequate final central PC pIOL vault. Sulcus-to-sulcus method provided higher vault predictability, although the difference between the 2 methods was not statistically significant.

An important issue for any refractive procedure in keratoconus is whether it could provide a cornea weakness, which could increase the risk for disease progressions. Ali et al. [41] carried out a comparative study to assess the changes in corneal biomechanics after PC pIOL implantation form normal and keratoconic patients. They found no significant changes in corneal hysteresis and corneal resistance factor after PC pIOL implantation, neither in normal eyes nor in keratoconic eyes. The authors pointed out that PC pIOL implantation in keratoconic patients could be safer than corneal refractive surgery, from a biomechanical point of view.

Despite the good visual and refractive outcomes reported, it should be taken into consideration that pIOLs can correct only spherical and cylindrical errors. It is very well documented that keratoconus induces a significant increase in HOAs [47, 48]. Keratoconus can have high levels of coma-like aberrations and spherical aberrations, among others [48], impacting negatively on the visual quality of the patients. These HOAs are not corrected by pIOLs.

The success of this pIOL implantation requires knowledge of the risk of progression of keratoconus, because of the progression of keratoconus leading to refraction change, and it could be a problem after pIOL implantation. Before surgery, a careful exploration should be performed to analyze whether signs of keratoconus progression are present.

### 2.3. Pseudophakic IOL

In keratoconic patients, the onset of cataract and/or presbyopia contributes to further decrease vision in already disabled patients. It is known that two of the most common human ocular afflictions are presbyopia and cataract [49]. Therefore, it seems evident that an important proportion of patients with keratoconus will develop age-related cataract and presbyopia. When a patient develops presbyopia or cataract, the corneal refractive surgery or phakic IOL implantation may not be the best option, being the replacement of the crystalline lens with a pseudophakic IOL the more proper approach.

Review of the literature was conducting using a combination of the following terms: “(keratoconus OR keratoconic) AND (intraocular lens OR cataract).” The databases used were PubMed, Web of Science, Embase, and Scopus. Once again, in this section, we excluded the studies in which this procedure was combined with other techniques. We found 18 articles, which met with our inclusion criteria.

Choosing the IOL power may be a challenge in keratoconus patients. Several articles focused on that. Celikkol et al. [50] suggested that videokeratography-derived *K* values might be more accurate than standard keratometry to calculate the IOL power. This study was carried out with only 2 eyes. Leccisotti [51] reported the outcomes after refractive lens exchange in a prospective noncomparative study, in which 34 eyes of 20 patients with stage I and II keratoconus were included. They concluded that refractive exchange in keratoconic eyes is a predictable procedure to correct myopia. However, 32% of the cases required an IOL exchange due to inaccurate IOL power calculation. Watson et al. [52] retrospectively reviewed the refractive outcomes of 92 eyes which underwent cataract surgery with implantation of a spherical IOL. They concluded that the actual *K* value with a target of low myopia is a proper option for spherical IOL choice for eyes with a mean *K* of ≤55 D. However, in the keratoconus eye, in which the mean *K* is higher than 55 D, the actual *K* values result in a large hyperopic error. Thebpatiphat et al. [53] compared the SRKI, SRKII, and SRK/T IOL formulas in patients with keratoconus and suggested that the SRKII formula might provide the most accurate IOL power in patients with mild keratoconus. However, in moderate and severe keratoconus, IOL calculations were less accurate and no differences in calculation formulas were found.

A source of error for IOL power calculation in keratoconic patients is the determination of the optical power of the cornea. Usually, the power of the cornea is estimated by considering only the radius of the anterior surface and a simulated refractive keratometric index. This estimation could lead to inaccuracies in the calculation of total corneal power in keratoconic eyes, where both the anterior and posterior surfaces of the cornea are affected. Tamaoki et al. [54] proposed to calculate the real corneal power values by taking both the anterior and posterior corneal curvature, using the current advanced technology, which provides the posterior corneal curvature. Camps et al. [55] introduced an approach for correcting the error in the estimation of corneal power in keratoconus, by means of a variable keratometric index that minimizes this error.

Despite the complexity of calculating IOL power in keratoconus, it should be considered that these patients may have better tolerance to defocus than healthy patients. So some residual refractive errors after IOL implantation can be better tolerated [56].

Regarding visual outcomes, several previous studies [57–64] have evaluated the results of replacement of lens (either by refractive lens exchange or cataract extraction) by a capsular bag toric intraocular lens (IOL). The first three articles [57–59] were case reports, which showed encouraging results. Subsequent case series studies [60–64] (from 12 to 23 eyes) reported a significant improvement in UDVA, CDVA, and refractive error (see Table 3). Recently, Meyer et al. [65] described 7 cases in which a supplementary sulcus-based toric IOL was implanted to compensate residual astigmatism in keratoconus patients with prior cataract surgery and spherical IOL implantation. They reported poor results in terms of rotational stability.

Two studies presented the outcomes with multifocal toric intraocular lens [66, 67]. Montano et al. [66] described two cases, a form fruste keratoconus and a stable keratoconus. Farideh et al. [67] evaluated the clinical results of toric intraocular trifocal IOL in 10 eyes (5 patients) with mild keratoconus. Both studies concluded that multifocal toric IOL provides satisfactory results in mild and stable keratoconus. However, this should be confirmed with further studies with a larger sample and a longer follow-up period.

Based on these previous studies, it seems that cataract extraction with a toric IOL implantation in patients with keratoconus may be a good option to restore the loss of visual quality caused by cataract. However, it is important to note that after cataract or clear lens extraction the risk of retinal detachment could be higher. Furthermore, it is important to bear in mind that even if the cataract is removed and the refractive error is significantly reduced after this procedure, the corneal abnormalities are still present. In Alió et al.'s study [62], the authors found that patients with more regular corneas obtained higher improvement of UDVA after surgery. Jaimes et al. [60] reported significant improvement in refractive error and UDVA, but not for CDVA. These studies reveal that although toric IOL implantation is a safe and effective option to improve refractive error and UDVA in cataractous or clear lens keratoconic patients, the corneal abnormalities may lessen the optimal restoration of the visual quality. Furthermore, the success of this procedure requires knowledge of the risk of progression of keratoconus, because of the progression of keratoconus leading to refraction change, and it could be a problem after IOL implantation. Before surgery, a careful exploration should be performed to analyze whether signs of keratoconus progression are present. However, a major risk factor for progression of keratoconus is young age; in fact, the onset of keratoconus is usually at puberty, and progression mainly occurs until the third of fourth decade of life [2]. Therefore, in this age group, the risk of keratoconus progression is minimal.

### 2.4. Intrastromal Corneal Ring Segments (ICRS)

The types of ICRS currently available can be grouped into two modalities: (1) ring segments: Intacs and Intacs SK (Addition Technology, Inc.) and Ferrara type (Keraring, Mediphachos, Inc. and Ferrara Ring (AJL, Spain)); (2) Full-ring: MyoRing (Dioptex, GmbH).

Intacs ICRS have an arc length of 150 degrees with an inner diameter of 6.8 mm. The thickness ranges from 0.25 to 0.45 mm in 0.5 mm steps. Intacs SK have an elliptical design; the optical zone is 6.0 mm and is available in two thicknesses (0.40 mm and 0.45 mm).

Ferrara-type ICRS has a triangular cross-section that induces a prismatic effect on the cornea. The apical diameter of ICRS is 5.0 mm ICRS (the flat basis width is 0.6 mm) or 6.0 mm (the flat basis width is 0.8 mm), with variable thicknesses (0.15 mm to 0.30 mm with 0.05 mm steps) and arc lengths (90, 120, 150, and 210 degrees).

The MyoRing is available in a diameter range of 5–8 mm and a thickness range of 0.2–0.4 mm in 0.02 mm increments. The width of the ring body is 0.5 mm. The anterior surface is convex, and the posterior surface is concave, with a radius of curvature of 8.0 mm.

ICRS implantation, both alone and in combination with corneal collagen cross-linking, can be considered to regularize the corneal shape and correct mild-moderate refractive error. Most of the studies report a reduction in the spherical equivalent of more than 2.00 D after ICRS implantation [68]. It should be considered that the shorter the arc length, the greater the effect on the refractive cylinder [69–72], whereas a 210° arc length provides corneal flattening and reduces the myopia [69, 73, 74]. An important issue is whether ICRS implantation halts or delays the progression of keratoconus. This issue will be discussed below in the combined procedure section.

## 3. Combined Procedures

The surgical procedures previously discussed may be interesting treatment options in order to correct spherical and cylindrical errors in stable keratoconus or keratoconus suspects. It is very well documented that keratoconus induces a significant increase in HOAs [47, 48]. Keratoconus can have high levels of coma-like aberrations and spherical aberrations, among others [48], impacting negatively on the visual quality of the patients. On the other hand, the progression of keratoconus leading to refraction and HOAs changes and it could be a problem after any of the surgical procedure previously described.

ICRS implantation and corneal collagen cross-linking have showed to be a safe and effective method to improve the keratoconus corneal shape, improving visual quality, and/or stop its progression [68, 75]. Keratoconus usually has associated ametropia, and after ICRS implantation or corneal CXL, most patients require contact lenses or spectacle to correct residual refractive error. The following sections review and discuss the results of the clinical studies in which ICRS implantation and corneal CXL were combined with other surgical procedures to correct the refractive error, improve visual quality, and/or stop or delay the keratoconus progression.

### 3.1. Double Procedure (ICRS + Intraocular Lens)

To the best of the author's knowledge, there are no studies that evaluated the ablative corneal refractive procedures to correct the residual refractive error after ICRS implantation. In this case, we adopted the following terms to perform the literature search: (keratoconus OR keratoconic) AND (intrastromal corneal ring segment OR myoring OR Keraring OR Ferrara ICRS OR icrs OR intacs) AND (lens OR ICL OR implantable Collamer OR phakic intraocular lens). In this section, we excluded the studies that include CXL as the third procedure, because these studies will be discussed in other sections. We found 10 articles, which met with our inclusion criteria.

#### 3.1.1. ICRS + Phakic IOL

The first study, which combined ICRS and an IOL, was a case report published in 2003. Colin and Velou [76] reported a case of Intacs implantation for keratoconus followed by the implantation of an AC pIOL to correct −8.25 D of residual error. 2 months after AC pIOL implantation, the UDVA and CDVA were 0.3 and 0.8 (decimal scale), respectively. Four years later, Kamburoğlu et al. [77] published a case report of a bilateral keratoconus in which Intacs were implanted in both eyes. Sixteen months after Intacs implantation, AC pIOL toric (Artisan toric) was inserted in both eyes to correct the residual myopic and astigmatic refractive errors. At five months, the UDVA was 0.6 and 0.5 in the right and left eyes, respectively, and CDVA 0.7 in both eyes. The first case series of this combined procedure was published in 2007 [78]. El-Raggal and Abdel Fattah, in a prospective study, evaluated the safety, efficacy, and stability of sequential Intacs insertion and AC pIOL (Verisyse pIOL) implantation in 8 keratoconus. The follow-up was 24 months. All eyes reached UDVA of 20/40 or better, and no eyes lost lines of CDVA compared to preoperative. The spherical equivalent at the last visit ranged from −1.75 D to +1.00 D. In a prospective nonrandomized study comparing simultaneous and sequential implantation of Intacs and AC pIOL (Verisyse), Moshirfar et al. [79] reported that combined insertion of Intacs and AC pIOL was safe and effective in all cases. They suggested that the outcomes of the simultaneous implantation of the Intacs and AC pIOL in 1 surgery were similar to the results obtained with sequential implantation using 2 surgeries. Ferreira et al. [80] retrospectively analysed the visual and refractive outcomes of 21 stable keratoconus who had ICRS implantation (Intacs and Intacs SK) followed by AC pIOL implantation (Artisan or Artiflex Toric) 6 months or later. For each of the two surgical procedures, both UDVA and CDVA improved significantly. After AC pIOL implantation, 61.9% of the eyes gained two or more lines of CDVA, and the spherical equivalent was within ±1.00 D of emmetropia in 90.5% of the eyes. Three studies [81–83] evaluated the results of combined ICRS and PC pIOL implantation in keratoconus. Coskunseven et al. [81] reported the results of three eyes who had undergone PC pIOL (toric ICL) implantation after Intacs implantation. The UDVA and CDVA were improved in the three cases, and all three were emmetropic within ±1.00 D after combined procedure. Alfonso et al. reported [82] for the first time the results of this combined procedure using Ferrara-type ICRS and performing the ICRS implantation using femtosecond laser. Their prospective study comprised 31 patients (40 eyes) with keratoconus who had ICRS implantation followed 6 months later by PC pIOL implantation with corneal relaxing incision. The mean spherical equivalent decreased from −9.66 ± 6.96 D preoperatively to −1.20 ± 1.33 D 6 months after PC pIOL implantation. At the end of the follow-up, 65% of eyes were within ±1.00 D of the desired refraction and 45% were within ±0.50 D. Of the 40 eyes analyzed, 3 eyes lost 1 line of monocular CDVA, 9 eyes had no change, 7 eyes gained 1 line, 10 eyes gained 2 lines, and 11 eyes gained more than 2 lines. The safety index (ratio of postoperative to preoperative monocular CDVA) 6 months after PC pIOL was 1.28, and the efficacy index (mean postoperative UDVA/mean preoperative CDVA) was 0.88. Navas et al. [83] in their retrospective study also concluded that combined treatment of keratoconus with ICRS and a pIOL or a toric pIOL was a safe and effective procedure for high refractive error correction induced by keratoconus in selected patients.

Based on the encouraging outcomes, it seems that sequential ICRS and pIOL implantation provides good visual and refractive outcomes, suggesting that the procedure is predictable for refractive correction of keratoconus. Combining ICRS with pIOLs may improve visual outcomes by combining the effects of ICRS in improving corneal shape with those of pIOLs in correcting spherical and cylindrical refractive errors. Any type of ICRS can be combined with any type of pIOL. ICRS and pIOL can be implanted simultaneously in one surgery, or sequentially in two surgeries. Although it has been suggested that both possibilities (simultaneous and sequential implantation) provide similar results [79], in sequential procedure, keratometry (K) readings can be taken after ICRS insertion, which theoretically ensures better prediction of the pIOL power before implantation.

#### 3.1.2. ICRS + Pseudophakic IOL

A sequential procedure has also been studied for the combined treatment of cataract and keratoconus [56, 84]. Alfonso et al. [56] reported the long-term results of sequential implantation of the Ferrara-type ICRS and IOL implantation in 70 eyes with keratoconus and cataract. The mean UDVA (logMAR scale) was 1.08 ± 0.24 preoperatively, 0.95 ± 0.31 six months after ICRS implantation, and 0.44 ± 0.29 six months after IOL implantation. The CDVA changed from 0.35 ± 0.23 (logMAR) before surgery to 0.28 ± 0.22 six months after Ferrara-type ICRS implantation and to 0.11 ± 0.16 six months after IOL implantation. Both the UDVA and CDVA were stable over the postoperative period of the second procedure. The spherical equivalent and the refractive cylinder declined steeply after IOL implantation and were also stable over the postoperative period. They concluded that sequential Ferrara-type ICRS and IOL implantation provides good visual and refractive outcomes, being an effective, safe, predictable, and stable procedure for the treatment of patients with keratoconus and cataract. Furthermore, the authors suggested that the ICRS implantation before IOL implantation could help in strengthening and/or reshaping the magnitude of the associated refractive error. The shape of the cornea may play an important role in the calculation of the IOL power. In less deformed corneas, more predictable results for the calculation of the IOL power should be expected. Hence, probably, the ICRS implantation before IOL implantation could help with IOL power calculations, because ICRS implantation improves the shape of the cornea, and it could help obtain a more accurate central corneal power and estimate the effective lens position in a better way. However, this hypothesis should be studied in futures studies.

It should be noted that the success of any sequential procedure (ICRS and pIOL or IOL) requires knowing when the refraction is stable after ICRS insertion and whether the keratoconus progression has been halted. There is debate and controversy about whether ICRS halts or delays the progression of keratoconus. Bedi et al. [85] reported on 105 eyes with Intacs over five years and found that 93 per cent of eyes with preoperative progressive keratoconus showed no postoperative progression. Long-term results of Ferrara-type ICRS implantation have been reported by Torquetti et al. in two studies [86, 87], one with a five-year follow-up [86] and the other with a ten-year follow-up [87]. They found that the refractive and visual outcomes were stable over the follow-up period. Progression of keratoconus at the moment of the surgery was an inclusion criterion in both studies. Fernández-Vega Cueto et al. [88] and Lisa et al. [74] also reported that Ferrara-type ICRS provides stable visual and refractive outcomes over 5- and 3-year follow-up, respectively. In Fernández-Vega Cueto's study [88], the results were stable even in young patients where the risk of keratoconus progression over the follow-up period is higher.

Vega-Estrada et al. [89, 90] carried out two studies in which they analysed the five-year long-term effects of ICRS implantation in both, nonprogressive keratoconus [89] and progressive keratoconus [90]. The authors concluded from the first study that the changes induced by ICRS are stable over a long period in patients with no evidence of keratoconus progression at the time of surgery [89]. In their second study [90], they examined the outcomes of ICRS implantation in young patients showing evidence of keratoconus progression and found that although ICRS implantation improved the visual and refractive outcomes in the short term, there was regression in the long term, which suggests that this procedure is not stable in young patients with evidence of keratoconus progression. However, it is important to note that this study had certain limitations. It was carried out on a total of 18 eyes, of which 13 eyes were implanted with Intacs ICRS (10 with the mechanical procedure and 3 with femtosecond), and 5 eyes were implanted with Ferrara-type ICRS (4 with femtosecond technology and 1 with the mechanical procedure). In addition, the keratoconus included in this study showed very strong progression of the disease (the mean *K* reading increased 3.17 D and the mean spherical equivalent 1.86 D in 6 months immediately prior to surgery).

Another important aspect to know whether any procedure is able to halt or delay the disease progression is how to document the keratoconus progression. According to Global Consensus on Keratoconus and Ectatic Diseases (2015), there is no consistent or clear definition of ectasia progression [91]. This panel defined progression by a consistent change in at least two of the following parameters: steepening of the anterior corneal surface, steepening of the posterior corneal surface, and/or thinning or changes in the pachymetric rate of change; nevertheless, the panel also agreed that specific quantitative data to define progression is lacking. Duncan et al. [92] published an interesting article in which showed the limitations of these clinical parameters to diagnose progression of keratoconus. They proposed a new software program to detect keratoconus progression.

In any case, it would be appropriate to conduct further studies because, as it will be discussed, a triple procedure (combining corneal cross-linking with ICRS implantation and intraocular lens or corneal refractive surgery) might be another alternative.

### 3.2. Corneal CXL

Corneal CXL is a minimally invasive procedure, which aims to increase the mechanical and biomechanical stability of the cornea. It is a photooxidative procedure consisting of combined application of riboflavin (vitamin B2) and ultraviolet A (UVA) light of 370 nm. The standard protocol for CXL was reported by Wollensak et al. [93] (it is currently known as the Dresden protocol for CXL). The corneal epithelium is removed, and riboflavin 0.1% solution is instilled for 30 minutes before UVA exposure. UVA irradiation is performed for an additional 30-minute period. High-fluency CXL and transepithelial CXL are variations of this technique, which aim to shorten the exposure time to UVA light and/or reduce patient discomfort and minimize potential complications.

There are many combination procedures such as corneal CXL and excimer laser surgery and corneal CXL and intraocular lens implantation reported in the literature aiming at therapeutic and refractive effect. The combined procedure of corneal CXL and ICRS implantation has been also studied in different articles. The main purpose of this combination is to improve the keratoconus corneal shape and halt its progression. As we explained in ICRS section, this combination can be used to correct mild-moderate refractive error, but not moderate or high refractive error. Because of this review focuses on the surgical approaches for therapeutic and refractive treatment of keratoconus, this combination has not been included in this section. The last section analyses the results of triple procedures (ICRS implantation and corneal CXL were combined with other surgical procedures).

#### 3.2.1. Double Procedure (Corneal CXL + Corneal Refractive Surgery)

In this section, the review of the literature was conducting using a combination of the following terms: “(keratoconus OR keratoconic) AND (crosslinking or CXL) AND (LASIK or PRK).” 25 studies were analysed. Kanellopoulos and Binder [94] were the first to attempt this combination procedure. They reported the case of a 26-year-old male patient with bilateral progressive keratoconus who had topography-guided PRK 12 months after corneal CXL in the left eye. 18 months after topography-guided PRK, the UDVA was 20/20 and CDVA was 20/15 with no evidence of disease progression. The authors concluded that this combined procedure seemed to be an effective method for the treatment of keratoconus. The authors also suggested that the nomogram for the laser ablation should be adjusted in patients who underwent corneal CXL. They pointed out that the more rigid cornea might have an ablation rate different from that of normal cornea. Subsequent cohort studies reported the outcomes of combined topography or wavefront-guided PRK and corneal CXL in keratoconic patients [95–112]. The results of these studies showed an UDVA after this combined procedure between 0.7 and −0.01 logMAR (with most of the studies where data are available reporting UDVA > 0.4 logMAR), CDVA ranging between 0.2 and −0.04 logMAR, and the spherical equivalent between −2.80 and +0.05 D. No evidence of disease progression was reported after a follow-up period ranging between 3 to 68 months. Furthermore, it has been reported in a retrospective analysis of 53 keratoconic eyes that distribution of epithelial thickness becomes more even after this combined treatment [113]. Fadlallah et al. [114] in a retrospective study reported that conventional PRK and corneal CXL were effective and safe options for correcting mild refractive error and improving visual acuity in early stable keratoconus. These favourable refracto-therapeutic results are accompanied by an improvement in the self-reported quality of life in keratoconus patients [110, 112, 115, 116]. It appears that cone location may have a significant impact on the outcomes of this combined procedure. A prospective, comparative case series study found that the visual results were superior in cone located within the central 2 mm zone than in cones located outside the central 2 mm zone [106].

Four prospective, case series studies compared the results of corneal CXL alone with combined simultaneous topography-guided PRK followed by corneal CXL [102, 104, 108, 111]. All of them agreed that the combined procedure provides better refractive and visual results than corneal CXL alone and similar results regarding postoperative stability.

Two prospective, comparative case series studies [96, 112] addressed the time interval between the 2 procedures (simultaneous versus sequential) and reached disparate results. In Kanellopoulos [96] study, the simultaneous approach provided superior visual and refractive results, whereas Abou Samra et al. [112] found comparable objective and subjective outcomes between the two options. Theoretically, simultaneous approach could provide more predictable refractive outcomes, because the ablation is performed before the corneal CXL. The ablation rate in a strengthened cornea by CXL is currently unknown. By contrast, it has been reported that the simultaneous procedure affects keratocyte density significantly [117, 118]. This finding could be a consequence of the sequence. The simultaneous procedure implies ablation of Bowman's membrane together with the epithelium before CXL treatment. So perhaps this could lead to a deeper penetration of riboflavin into the cornea [117, 118]. Further studies are needed to know which is the most effective and safe procedure.

Important considerations for this combined procedure are ablation depth and postoperative corneal thickness. Most of the studies recommended a maximum ablation depth of 50 *μ*m and a minimal postoperative corneal thickness of no less than 350 *μ*m. So, in keratoconus cases with a moderate-high ammetropia and/or thin cornea, this combined procedure would not be capable to correct the full refractive preoperative error.

#### 3.2.2. Double Procedure (Corneal CXL + Intraocular Lenses)

The combination of corneal CXL and IOL implantation is another alternative to stabilize the keratoconus and correct the residual refractive error. In this case, we adopted the following terms to perform the literature search: “(keratoconus OR keratoconic) AND (crosslinking or CXL) AND (lens OR ICL).”

*(1) Corneal CXL + Phakic IOL*. The combination of corneal CXL and PC pIOL (toric ICL) implantation was first reported by Kymionis et al. [119]. A 29-year-old woman with progressive keratoconus underwent toric PC pIOL implantation 12 months after corneal CXL. At 3 months, the UDVA rose from counting fingers to 20/40 and the CDVA improved from 20/100 to 20/30. Furthermore, no intraoperative or postoperative complications were observed. The authors concluded that this combined procedure in a 2-step procedure seemed to be an effective method for correcting keratoconus in patients with high myopia and astigmatism. Favourable outcomes have since been reported in keratoconic patients who underwent this combined procedure (corneal CXL and PC pIOL implantation) [37, 120–122], with all studies reporting that PC pIOLs or toric PC pIOLs were a predictable, safe, and effective way to correct refractive error in patients with keratoconus following corneal CXL. Fadlallah et al. [120] evaluated the safety and clinical outcomes of toric PC pIOL implantation 6 months after corneal CXL in 16 eyes of 10 patients. Six months after toric PC pIOL implantation, the mean of spherical equivalent decreased from −7.24 ± 3.53 to −0.89 ± 0.76 D and the mean cylinder dropped from 2.64 ± 1.28 to −1.16 ± 0.64 D. Shafik Shaheen et al. [121] reported a 3-year-long-term clinical study to assess the predictability, efficacy, safety, and stability in patients who received a toric PC pIOL after corneal CXL in early stage keratoconus. The study included 16 eyes. The mean spherical equivalent after toric PC pIOL was less than −0.25 D. Antonios et al. [122] evaluated the long-term safety and clinical outcomes of progressive keratoconus patients who had sequential corneal CXL followed by toric PC pIOL implantation after 6 months. The study included 30 eyes. 6 months after corneal CXL, no change in visual acuity or refraction was obtained. Twelve months after toric PC pIOL implantation, the mean spherical equivalent improved from −6.96 ± 3.68 D preoperatively to −0.83 ± 0.76 D. The mean cylinder, in turn, varied from 2.95 ± 1.40 D to 1.03 ± 0.60 D. Both UDVA and CDVA improved after toric PC pIOL implantation, and the values were maintained during the follow-up.

Implantation of the AC pIOL following corneal CXL is another possibility to correct the refractive error. Izquierdo et al. [123] in prospective study evaluated the safety, efficacy, and stability of the AC pIOL (Artiflex) implantation 6 months after corneal CXL in progressive keratoconus. The results of this case series study showed a significant improvement in visual acuity, keratometry, and refractive error, 6 months after AC pIOL implantation, and no intraoperative or postoperative complications were reported. Güell et al. [124] retrospectively reported long-term outcomes of combined corneal CXL and toric AC pIOL (Artiflex or Artisan) implantation, concluding that this is a safe and effective approach to correct myopic astigmatism in progressive mild to moderate keratoconus.

*(2) Corneal CXL + Pseudophakic IOL*. Corneal CXL followed by phacoemulsification with IOL implantation (either by refractive lens exchange or cataract extraction) has also been studied [125, 126]. Spadea et al. [125] described two patients with cataract and progressive keratoconus who underwent a 2-stage treatment: first corneal CXL followed by phacoemulsification with IOL implantation. The time interval between two procedures was at least 6 months. In both cases, UDVA and CDVA improved after IOL implantation. In a prospective study, Abou Samra et al. [126] evaluated the outcomes of a corneal CXL followed by phacoemulsification with toric IOL implantation in 9 eyes diagnosed with progressive keratoconus. The preoperative spherical equivalent was −8.11 ± 1.76 D improving to −0.91 ± 0.77 D 12 months postoperatively. The UDVA (logMAR scale) rose from a preoperative 1.43 ± 0.51 preoperatively to a 12-month postoperative 0.30 ± 0.09 logMAR. The CDVA, in turn, varied from 0.34 ± 0.12 to 0.24 ± 0.13. From these outcomes, the authors concluded that this two-stage approach was a safe and effective procedure in terms of keratometric stability and visual and refractive outcomes in patients with keratoconus.

Based on these encouraging outcomes, it seems that corneal CXL and IOL implantation (phakic and pseudophakic) provides good visual and refractive outcomes, suggesting that this combined procedure might be an effective procedure for stabilizing the disease and improving visual and refractive outcomes. Despite these good outcomes, it should be taken into consideration that this combination could stabilize the disease, but only correct spherical and cylindrical errors. Patients with poor CDVA because of irregular astigmatism and/or HOAs would require an additional procedure to improve the corneal shape.

#### 3.3. Triple Procedure (ICRS AND Corneal CXL AND…)

A 3-stage procedure has been proposed to halt the ectatic process, improve the corneal shape and visual acuity, and minimize the residual refractive error. Five studies analysed the efficacy and safety of the triple procedure: corneal CXL, ICRS implantation, and PRK [123–132]. All of these studies reported significant improvement in visual acuity, refraction, and corneal shape. This triple procedure can be performed in 2-stage ICRS implantation followed by simultaneous PRK and corneal CXL [127–130] or in a three-step procedure ICRS implantation followed by corneal CXL and PRK 6 months later [131, 132].

Three studies evaluated the outcomes of patients treated with the 3-stage procedure: ICRS implantation + corneal CXL + phakic IOL implantation. In a prospective case series study, Coşkunseven et al. [133] evaluated this 3-stage procedure in 14 eyes. The time interval among surgeries was 6 months. The mean manifest refraction spherical equivalent decreased from −16.40 ± 3.56 D to 0.80 ± 1.02 D after the 3 combined treatments. The refractive outcomes were stable in all eyes over 12 months of follow-up. There was a significant improvement in CDVA after the 3-stage procedure. The authors concluded that this combined 3-stage approach was effective in improving functional vision and reducing disease progression in keratoconic eyes. Jarade et al. [134] retrospectively analysed 11 keratoconus who had 3-step ICRS implantation followed by CXL and then toric PC pIOL implantation. The time interval between ICRS implantation and CXL was 4 weeks and between CXL and toric PC pIOL implantation was at least 6 months. The combined procedure resulted in significant improvements in UDVA and CDVA. The spherical equivalent decreased from −9.70 ± 3.1 D to −0.58 ± 1.01 D 6 months after toric PC pIOL implantation. The authors also concluded that the 3-step procedure was safe, effective, and predictable in the treatment of selected cases of keratoconus. Dirani et al. [135] retrospectively examined the results of this triple procedure in 11 eyes with moderate to severe keratoconus. They also found that the UDVA, CDVA, and refractive error improved significantly after toric PC pIOl implantation.

Sideroudi et al. [136] assessed the impact of corneal CXL on the material properties of ICRS. They found that an amount of riboflavin solution was absorbed into the samples of ICRS analyzed after CXL procedure. El-Raggal [137] evaluated the effect of corneal CXL on femtosecond laser channel creation for ICRS implantation. The results showed that the laser power must be modified after CXL. The author suggested that channel dissection and ICRS implantation should be performed before or concurrent with CXL. These findings should be taken into consideration when CXL is combined with ICRS implantation.

Assaf and Kotb [138] proposed another possibility of triple procedure: simultaneous PRK and corneal CXL followed by AC pIOL implantation. The time interval between simultaneous PRK and CXL was 2–4 months. The study included 22 eyes. The mean spherical equivalent was reduced from −9.08 ± 2.5 D preoperatively to −0.69 ± 0.67 D postoperatively. The UDVA improved from 1.24 ± 0.49 to 0.37 ± 0.08 logMAR and CDVA from 0.69 ± 0.3 to 0.35 ± 0.01. The authors concluded that this triple procedure improved and stabilized visual performance in patients with keratoconus. They proposed that large-scale studies with a longer follow-up are needed to assess this approach.

## 4. Conclusion

**Figure 1:**
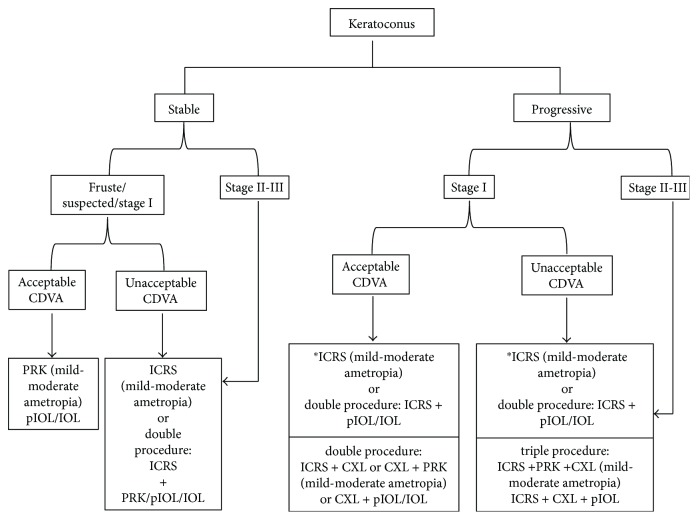
Decision tree treatment considering the stability or progression, the corrected distance visual acuity (CDVA), and the refractive error. PRK = photorefractive keratectomy; pIOL = phakic intraocular lens; IOL = pseudophakic intraocular lens; ICRS = intrastromal corneal ring segments; CXL = corneal collagen cross-linking. ^∗^If keratoconus is stable after ICRS implantation.

Keratoconus patients present significantly impaired quality of life that deteriorates as the disease progresses [4, 5]. The treatment used as well as the method to correct the refractive error of these patients may also influence on the impact of the disease on their quality of life [6, 12, 115, 116]. Keratoconus was historically once considered a contraindication to refractive surgery. However, the range of refractive procedures available now, as well as the advanced technology to accurately diagnose and follow the keratoconus, opens a new frontier in the keratoconus treatment by means of refractive surgery. Generally, the visual rehabilitation for keratoconic corneas requires addressing three concerns: halting the ectatic process, improving corneal shape, and minimizing the residual refractive error. The treatment would depend, among others factors, on each of these concerns and its influence on the disease and quality of vision of the patient. That is, keratoconus is progressing or not, the cornea shape is very irregular with high level of HOAs and poor CDVA or not, and finally the degree of associated ametropia. In this sense, corneal CXL can slow down or halt the disease progression, ICRS implantation can improve the corneal shape and hence the visual quality and reduce the refractive error, PRK can correct mild-moderate refractive error, and IOL can correct from low to high refractive error associated with keratoconus. Any of these surgical options can be performed alone or combined with the other techniques depending on what the case requires (strengthening or reshaping associated refractive error).  Figure 1 shows a decision tree treatment considering the stability or progression, the CDVA, and the refractive error.

Basing on the results published up to now, it seems that the surgical techniques (both, when used alone and in combination) provide safe and effective results for the refracto-therapeutic treatment in selected cases of keratoconus. However, all these techniques should be considered carefully as the follow-up periods of the relevant studies are relatively short. Most of these studies are case series or retrospective analysis, which include small number of cases.

In summary, although it could be considered that the surgical option for the refracto-therapeutic treatment of the keratoconus is a reality, controlled, randomized studies with larger cohorts and longer follow-up periods are needed to determinate which refractive procedure and/or sequence are most suitable for each case.

## Figures and Tables

**Table 1:** Summary of visual and refractive outcomes of the anterior chamber phakic intraocular lens implantation in keratoconic eyes.

Author/year	Level of evidence [13]	pIOL	Eyes (*n*)	KC stage	Follow-up (months)	Mean change from preoperative to last follow-up visit				Predictability SE		Complications
						UDVA	CDVA	Sphere (D)	Cylinder (D)	Within ±0.5 D (%)	Within ±1.00 D (%)	
Leccisotti and Fields [27]	IV	Angle supported	12	I to II	16.5 (range 12 to 24)	—	0.14 (decimal)	12.4	0.33	67	100	pIOL rotation (8.3%) Pupil ovalization (25%)
Budo et al. [28]	IV	Artisan toric	6	—	6	—	0.2 (decimal)	11.3	2.42	20	66.67	—
Moshirfar et al. [29]	IV	Verisyse	2	—	5 and 2	0.58 (decimal)	0.08 (decimal)	13.4	0.62	0	50	—
Venter [30]	IV	Artisan	18	—	From 6 to 12	—	0.20 (decimal)	4.67	3.93	61.1	83.3	Keratoconus progression (5.56%)
Sedaghat et al. [31]	IV	Artisan	16	—	14.2 (range 6 to 28)	0.71 (decimal)	0.14 (decimal)	12.47	0.87	33.33	53.33	—
Kato et al. [32]	IV	Artisan/Artiflex	36	—	12	1.32 (logMAR)	—	—	1.82	63.6	83.6	—

**Table 2:** Summary of visual and refractive outcomes of the posterior chamber phakic intraocular lens implantation in keratoconic eyes.

Author/year	Level of evidence [13]	pIOL	Eyes (*n*)	KC stage	Follow-up (months)	Mean change from preoperative to last follow-up visit				Predictability SE		Complications
						UDVA	CDVA	Sphere (D)	Cylinder (D)	Within ±0.5 D (%)	Within ±1.00 D (%)	
Alfonso et al. [33]	IV	ICL	25	—	12	—	0.05 (decimal)	8.6	0.71	84	100	No
Kamiya et al. [34]	IV	Toric ICL	2	—	3	0.79 (decimal	0.4 (decimal)	9.1	3.25	50	100	No
Alfonso et al. [35]	IV	Toric ICL	30	I and II	12	0.66 (decimal)	0.07 (decimal)	3.62	3.07	93.3	100	No
Kamiya et al. [36]	IV	Toric ICL	27	I and II	6	1.6 (logMAR)	0.04 (logMAR)	—	2.47	85	96	No
Kurian et al. [37]	IV	ICL and toric ICL	10	I to III	6	0.52 (decimal)	0.11 (decimal)	5.20	2.15	30	70	—
Hashemian et al. [38]	IV	Toric ICL	22	I to III	6	—	0.22 (decimal)	3.8	1.54	68.2	90.9	No
Kamiya et al. [39]	IV	Toric ICL	21	I and II	36	1.52 (logMAR)	0.05 (logMAR)		2.59	67	86	No

**Table 3:** Summary of visual and refractive outcomes of the pseudophakic intraocular lens implantation in keratoconic eyes.

Author/year	Level of evidence [13]	IOL	Eyes (*n*)	KC stage	Pre-UDVA	Pre-CDVA	Follow-up (months)	Spherical equivalent (D)	Post-UDVA	Post-CDVA
Jaimes et al. [60]	IV	AcrySof toric SN60TT	19	Keratoconus suspect; keratoconus and PMD	1.35 ± 0.36 (logMAR)	0.28 ± 0.55 (logMAR)	7.8 (range 3 to 31)	0.46 ± 0.10	0.29 ± 0.23 (logMAR)	0.11 ± 0.12 (logMAR)
Nanavaty et al. [61]	IV	AT TORBI 709 M	12	Mild to moderate	1.30 ± 0.50 (logMAR)	1.00 ± 0.80 (logMAR)	9 (range 3 to 25)	0.10 ± 0.60	0.30 ± 0.30 (logMAR)	0.10 ± 0.10 (logMAR)
Alió et al. [62]	IV	AcrySof IQ toric	17	—	1.33 ± 0.95 (logMAR)	0.32 ± 0.38 (logMAR)	9.1 (range 6 to 15)	−0.62 ± 0.97	0.32 ± 0.38 (logMAR)	0.20 ± 0.36 (logMAR)
Hashemi et al. [63]	IV	AcrySof toric SN60TT	23	Mild	0.90 ± 0.64 (logMAR)	0.28 ± 0.10 (logMAR)	3	−0.58 ± 0.95	0.27 ± 0.18 (logMAR)	0.16 ± 0.09 (logMAR)
				Moderate	1.00 ± 0.48 (logMAR)	0.51 ± 0.52 (logMAR)		−0.34 ± 0.90	0.34 ± 0.19 (logMAR)	0.18 ± 0.12 (logMAR)
				Severe	1.30 ± 0.00 (logMAR)	0.83 ± 0.55 (logMAR)		0.50 ± 0.58	0.38 ± 0.29 (logMAR)	0.35 ± 0.13 (logMAR)
Kamiya et al. [64]	IV	AcrySof IQ toric (SN6AT)	19	I and II	1.14 ± 0.50 (logMAR)	0.27 ± 0.45 (logMAR)	3	−1.64 ± 1.41	0.46 ± 0.33 (logMAR)	−0.01 ± 0.09 (logMAR)
